# Proportional Mortality due to Heart Failure and Ischemic Heart Diseases in the Brazilian Regions from 2004 to 2011

**DOI:** 10.5935/abc.20160119

**Published:** 2016-09

**Authors:** Eduardo Nagib Gaui, Carlos Henrique Klein, Glaucia Maria Moraes de Oliveira

**Affiliations:** 1 Programa de Pós Graduação em Cardiologia - Universidade Federal do Rio de Janeiro, Rio de Janeiro, RJ - Brazil; 2 Escola Nacional de Saúde Pública - Fiocruz, Rio de Janeiro, RJ - Brazil

**Keywords:** Heart failure/mortality, Myocardial Ischemia/mortaity, Death Certificates, Epidemiology, Brazil

## Abstract

**Background:**

Heart failure (HF) and ischemic heart diseases (IHD) are important causes of death in Brazil.

**Objective:**

To assess proportional mortality (PM) due to HF and IHD as underlying causes stratified by sex and age groups in the Brazilian geoeconomic regions from 2004 to 2011.

**Methods:**

Data from death certificates were obtained in the DATASUS site under the following International Statistical Classification of Diseases and Related Health Problems codes, 10^th^ Revision: 1) from chapter IX: I20 to I24 for acute IHD, I25 for chronic IHD, and I50 for HF; and 2) from chapter XVIII, for ill-defined causes (IDC).

**Results:**

Proportional mortality due to HF increased with age in both sexes and all regions, the highest percentages being found among elderly women. Among men, the highest percentages were observed in the West-Central region up to the ninth decade, but, among the eldest individuals, the highest percentages were identified in the Southern region. Among women, the regions did not differ up to the age group of 70-79 years, although the West-Central region took the lead from 50 to 79 years; however, from the age of 80 years on, the Southern region showed the highest PM due to HF. Proportional mortality due to acute IHD in all Brazilian regions and in both sexes increased up to the age group of 60-69 years, from which it decreased. Among men, the Southeastern region had the highest percentages in the age group of 50-59 years, while women had lower PM due to acute IHD than men in all regions. In both sexes, PM due to chronic IHD increased with age in the Southern and Southeastern regions, which did not happen in the others, while the Southern region had the highest rate of all regions for all age groups.

**Conclusions:**

Regional differences were more prominent at more advanced ages, especially when deaths due to IDC were excluded.

## Introduction

Diseases of the circulatory system (DCS) are important causes of death in Brazil. The World Health Organization provides a classification of diseases, the International Statistical Classification of Diseases and Related Health Problems (ICD),^1^ whose purpose is to permit the systematic recording analysis, interpretation and comparison of mortality and morbidity data. 

In the death certificate model used in Brazil, the 49^th^ field is intended to record the causes of death. This field is divided into two parts. Part I has four rows (a, b, c, and d), where all diseases or injuries that contributed to or produced death, or the circumstances of the accident or violence that produced these injuries must be registered. Line a should be used to the immediate cause. Lines b, c, and d should be used to the causes that led to the immediate cause (line a), line d being reserved for the underlying cause of death. In Part II any significant morbid condition that could have influenced unfavorably the evolution of the disease process is recorded, thereby contributing to the patient's death, without, however, relating to the disease or medical condition that directly caused the death. These causes are called contributing causes. It should be noted that even if there is a line for stating the underlying cause of death, the selection of that cause is defined by specific rules set in the 10^th^ Revision of ICD (ICD-10).

Chapter IX of ICD-10 provides the alphanumeric codes to classify DCS, which were identified as the underlying cause of death in 335,177 of the 1,169,966 death certificates issued in 2011 in Brazil, corresponding to 29% of the total deaths in that year.^2^

Of the DCS, the following stand out: heart failure (HF) and acute and chronic ischemic heart diseases (IHD). A large number of survivors of acute IHD episodes progress to HF, which is the final stage of several heart diseases.^3,4^

Heart failure accounts for high morbidity and mortality, and its prevalence tends to increase with both population aging and the increased survival of patients experiencing acute coronaryevents.^5^ In Brazil, HF has been reported as an important cause of hospitalization, mainly after the age of 60 years.^6^

Ischemic heart diseases accounted for 31% of the deaths due to DCS in Brazil in 2011,^2^ and remain as the major cause of death in adults in Latin America.^7^ In Brazil, mortality rates due to IHD, standardized according to age, showed a mild declining trend from 1996 to 2011.^8^

This study aimed at assessing proportional mortalities due to HF and IHD, selected as underlying causes of death, stratified by sex and age, in the Brazilian geoeconomic regions from 2004 to 2011.

## Methods

Data from death registries, comprising death certificates, of the Brazilian states from 2004 to 2011 were obtained in the Brazilian Health System database (DATASUS)^2^. The number of death certificates from each of the five Brazilian geoeconomic regions (Northern, Northeastern, West-Central, Southeastern and Southern) was calculated by adding the number of death certificates of the states forming each Brazilian region. The study period (from 2004 to 2011) was chosen based on the following: it has been only since 2004 that all causes of death notified by certifiers using all lines of the document [parts I (from a to d) and II] have been recorded in all Brazilian states; 2011 was the last year available at the time of data collection for this study.

The electronic death registries used the mortality classification of ICD-10, from which the following codes were selected: 1) from chapter IX: I20 to I24 for acute IHD; I25 for chronic IHD; and I50 for HF; and 2) from chapter XVIII, the codes for ill-defined causes (IDC).^1^ The age groups (in years) studied were as follows: 0-39; 40-49; 50-59; 60-69; 70-79; 80-89; and 90 or older.

Proportional mortality is a measure of the importance of a specific cause of death in relation to all causes of death within the same population group. This study assessed proportional mortalities due to HF, acute IHD, chronic IHD, and IDC selected as underlying causes of death, stratified by sex and age, in the Brazilian geoeconomic regions (Tables 1 and 2).

**Table 1:** Proportional mortality (%) due to heart failure, acute or chronic ischemic heart diseases and ill-defined causes as underlying causes of death in the Brazilian geoeconomic regions stratified by age group in the male sex from 2004 to 2011

Cause	Region					
Age group	Northen	Northestern	West-Central	Southeastern	Southern	Brazil
**HF**						
0-39	0.4	0.4	0.5	0.3	0.2	0.4
40-49	1.3	1.2	1.4	1.0	0.9	1.1
50-59	1.9	1.9	2.4	1.5	1.4	1.6
60-69	3.0	2.6	3.2	2.2	2.1	2.4
70-79	3.5	3.3	3.7	2.9	3.1	3.1
80-89	4.4	4.1	4.7	3.7	4.5	4.0
90ou+	4.1	4.9	4.9	4.4	5.9	4.8
Total	2.1	2.3	2.5	2.1	2.2	2.2
**Acute IHD**						
0-39	0.8	1.1	1.1	1.3	1.1	1.2
40-49	5.8	6.6	7.0	7.4	7.1	7.0
50-59	9.2	10.3	11.2	11	10.7	10.7
60-69	9.5	11.5	11.3	11.5	11.3	11.3
70-79	8.7	9.9	9.7	9.8	9.8	9.8
80-89	6.5	7.7	7.0	7.8	7.6	7.6
90ou+	5.2	6.2	5.1	5.6	5.8	5.8
Total	5.4	6.9	7.1	8.0	7.9	7.5
**Chronic IHD**						
0-39	0.0	0.1	0.1	0.1	0.1	0.1
40-49	0.3	0.4	0.9	0.9	1.0	0.8
50-59	0.5	0.9	1.7	1.7	1.9	1.5
60-69	0.7	1.2	2.1	2.2	2.5	2.0
70-79	0.8	1.2	2.0	2.3	2.8	2.1
80-89	0.7	1.1	1.8	2.3	2.9	2.0
90ou+	0.6	0.9	1.8	2.3	2.6	1.7
Total	0.4	0.8	1.3	1.6	2.0	1.4
**IDC**						
0-39	8.5	5.5	3.1	5.8	3.3	5.4
40-49	13.0	9.2	5.0	9.4	6.1	8.8
50-59	13.7	9.7	5.2	8.7	5.8	8.5
60-69	14.9	10.4	4.8	8.0	5.3	8.1
70-79	16.7	11.8	4.6	7.2	5.0	8.1
80-89	20.4	14.9	5.3	7.5	5.8	9.7
90ou+	28.9	21.3	8.9	11.1	10.6	15.4
Total	13.6	10.4	4.6	7.7	5.3	8.2
**N**						
0-39	105,308	343,734	86,283	433,740	141,069	1,110.134
40-49	28,015	115,165	34,803	232,483	74,713	485,179
50-59	33,372	141,644	43,708	339,116	111,350	669,190
60-69	39,834	176,257	52,799	398,437	145,124	812,451
70-79	45,588	209,710	57,177	462,905	167,670	943,050
80-89	31,220	185,762	37,783	324,647	111,796	691,208
90ou+	10,272	71,998	11,102	83,886	26,570	203,828
Total	293,609	1,244.270	323,655	2,275.214	778,292	4,915.040

ICD-10: International Statistical Classification of Diseasesand Related Health Problems - 10^th^ revision. ICD-10: codes: from Chapter IX for HF - 150, for acute IHD - 120-124, and for chronic IHD - 125; and from Chapter XVIII for IDC. HF: heart failure; IHD: acute or chronic ischemic heart diseases; IDC: ill-defined causes.

**Table 2:** Proportional mortality (%) due to heart failure, acute or chronic ischemic heart diseases and ill-defined causes as underlying causes of death in the Brazilian geoeconomic regions stratified by age group in the female sex from 2004 to 2011

Cause	Region					
Age group	Northen	Northestern	West-Central	Southeastern	Southern	Brazil
**HF**						
0-39	0.6	0.7	0.5	0.5	0.4	0.6
40-49	1.2	1.6	1.6	1.2	1.2	1.3
50-59	1.9	2.3	2.5	1.9	1.8	2.0
60-69	2.8	2.8	3.3	2.6	2.8	2.8
70-79	3.4	3.3	4.4	3.4	4.2	3.6
80-89	3.8	4.0	5.1	4.3	5.8	4.5
90 or +	4.3	4.6	5.8	4.9	7.4	5.2
Total	2.4	2.9	3.3	3.0	3.8	3.1
**Acute IHD**						
0-39	0.7	1.1	1.0	1.2	1.0	1.1
40-49	4.7	7.1	6.0	6.3	6.3	6.4
50-59	6.8	9.2	7.8	8.2	8.2	8.4
60-69	7.7	10.1	9.1	9.4	9.4	9.5
70-79	7.1	9.2	8.2	9	9.2	9
80-89	6.0	7.5	6.6	7.3	7.6	7.3
90 or +	4.8	5.9	4.9	5.8	6	5.8
Total	4.9	7.2	6.3	7.3	7.5	7.1
**Chronic IHD**						
0-39	0.0	0.1	0.1	0.1	0.1	0.1
40-49	0.2	0.5	1.0	0.8	0.9	0.7
50-59	0.4	0.8	1.4	1.4	1.6	1.2
60-69	0.6	1.0	1.8	1.9	2.2	1.7
70-79	0.8	1.2	1.8	2.1	2.7	1.9
80-89	0.7	1.1	1.8	2.2	3.0	2.0
90 or +	0.7	1.0	1.9	2.4	3.2	2.0
Total	0.5	0.9	1.4	1.7	2.2	1.5
**IDC**						
0-39	9.8	7.3	3.4	6.1	3.7	6.3
40-49	11.3	8.9	4.3	7.4	4.5	7.3
50-59	11.8	8.8	3.6	7.0	4.4	7.0
60-69	13.4	9.6	3.6	6.8	4.3	7.2
70-79	15.7	11.2	3.7	6.9	4.7	7.7
80-89	20.1	14.5	5.0	7.7	6.1	9.4
90 or +	29.8	21.7	9.5	11.2	11.0	14.8
Total	14.8	11.8	4.3	7.5	5.4	8.4
**N**						
0-39	49,782	146,364	36,437	184,665	59,689	476,937
40-49	14,073	60,516	17,437	119,553	37,702	249,281
50-59	19,309	92,027	25,253	192,631	62,242	391,462
60-69	25,177	134,654	34,437	269,106	92,995	556,369
70-79	32,284	187,067	43,297	400,302	138,749	801,699
80-89	28,163	197,554	36,886	415,142	139,630	817,375
90ou+	14,674	101,902	15,540	174,025	53,005	359,146
Total	183,462	920,084	209,287	1,755,424	584,012	3,652,269

ICD-10: International Statistical Classification of Diseasesand Related Health Problems - 10^th^ revision. ICD-10: codes: from Chapter IX for HF - 150, for acute IHD - 120-124, and for chronic IHD - 125; and from Chapter XVIII for IDC. HF: heart failure; IHD: acute or chronic ischemic heart diseases; IDC: ill-defined causes.

**Figure 1:**
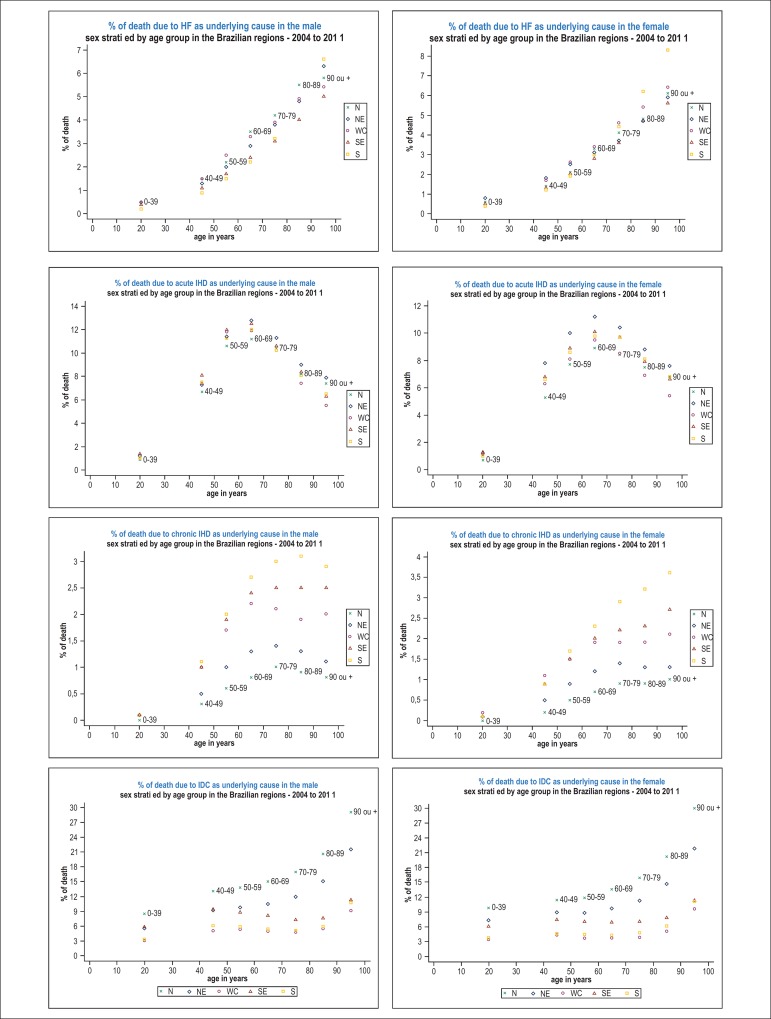
Proportional mortality due to heart failure (HF), acute and chronic ischemic heart diseases (IHD) as underlying causes of death, excluding deaths of ill-defined causes (IDC), and due to IDC as underlying cause of death, in the Brazilian geoeconomic regions, stratified by age group and sex, from 2004 to 2011. HF: heart failure; IHD: ischemic heart disease; IDC: ill-defined causes; NO: Northern; NE: Northeastern; WC: West-Central; SE: Southeastern; SO: Southern ICD-10: International Statistical Classification of Diseases and Related Health Problems - 10^th^ revision. ICD-10 codes: from Chapter IX for HF - I50, for acute IHD - I20-I24, and for chronic IHD - I25; and from Chapter XVIII for IDC.

The graphs of Figure 1 show the proportional mortalities due to HF and acute and chronic IHD, except for the deaths whose causes were encoded as ill-defined, stratified by sex and age groups in the Brazilian geoeconomic regions. Thus, the calculation of the proportional mortalities shown in Figure 1 considered only deaths due to defined causes in their denominators.

Stata statistical software, version 12, was used to calculate the percentages and to elaborate the graphs (Stata Corporation, College Station, Texas, USA).^9^

## Results

In Brazil, 8,597,955 deaths were registered from 2004 to 2011. The ICD-10 codes of the underlying causes of death retrieved from death certificates were as follows: HF, 2.6%; acute IHD, 7.3%; and chronic IHD, 1.4%. In that period, 8.3% of the death certificates had IDC as the underlying cause of death.

The distribution of proportional mortalities due to HF in the Brazilian regions was as follows, the highest figures found in the Southern region, and the lowest, in the Northern region: Southern region, 2.9%; West-Central, 2.8%; Northeastern and Southeastern, 2.5% each; and Northern region, 2.2%. Deaths due to acute IHD were proportionally more frequent in the Southern and Southeastern regions (7.7% and 7.6%, respectively), followed by the Northeastern, West-Central and Northern regions (7.0%, 6.8% and 5.2%, respectively). Regarding chronic IHD, the distribution of proportional mortalities was as follows: Southern, 2.1%; Southeastern, 1.7%; West-Central, 1.4%; Northeastern, 0.8%; and Northern, 0.4%.

Regarding IDC, the distribution of proportional mortality according to the Brazilian regions was as follows, with the highest figures observed in the Northern region: Northern, 14.1%; Northeastern, 11.0%; Southeastern, 7.7%; Southern, 5.3%; and West-Central, 4.5%.

Table 1 shows the proportional mortality due to HF, acute or chronic IHD, and IDC as underlying causes of death in the Brazilian regions according to the age group in men from 2004 to 2011. Table 2 shows the same for women.

The proportional mortality due to HF increased with age increase in all Brazilian regions in both sexes (Tables 1 and 2), although such increase was not observed in men aged 90 years or older in the Northern region. In the male sex, the highest percentages were observed in the West-Central region up to the age group of 80-89 years, but, among the eldest individuals, the highest percentages were observed in the Southern region. However, the Southern region had the lowest proportional mortalities up to the age group of 60-69 years. From that age onward, while the Southeastern region continued to show the lowest proportional mortalities up to the age group of 80-89 years, the Southern region stood up with the most notable increase of all regions in the male sex (Table 1). In the female sex, no significant difference was identified among the regions up to the age group of 70-79 years, although the West-Central region took the lead from 50 years to 79 years; however, from the age of 80 years on, the Southern region showed the highest proportional mortalities due to HF (Table 2).

The proportional mortality due to acute IHD in all Brazilian regions and in both sexes increased up to the age group of 60-69 years, from which it decreased (Tables 1 and 2). But women still had lower proportional mortality due to acute IHD than men did in all regions. However, for both sexes, the differences between regions in almost all age groups were not significant, except for the Northern region, which always had the lowest proportional mortalities (Tables 1 and 2).

The proportional mortalities due to chronic IHD were lower than those due to the other selected causes (Tables 1 and 2). For both sexes, mortality increased with age in the Southern and Southeastern regions, but not in the other regions. The proportional mortality due to chronic IHD in the Southern region was higher than in the other regions for all age groups in the female sex (Table 2); in the male sex, however, this was observed only for the age of 50 years and older (Table 1). That was followed in decreasing order and for both sexes by the regions: Southeastern, West-Central, Northeastern and Northern (Tables 1 and 2).

The proportional mortalities due to IDC increased progressively with age in the Northern and Northeastern regions for both sexes (Tables 1 and 2). In addition those two regions stood out because of very high percentages as compared with the others. The West-Central region had the lowest percentages of death for all age groups and both sexes. It is worth noting that the oldest individuals (90 years or older) had the highest proportional mortalities for both sexes and all regions (Tables 1 and 2).

The graphs of Figure 1 show the percentages of death due to HF, acute and chronic IHD as underlying causes in the Brazilian regions, stratified by age group and sex, from 2004 to 2011. The calculation of the proportional mortalities shown considered in their denominators only deaths due to defined causes; those due to IDC were excluded.

The proportional mortalities due to HF, excluding IDC, increased with age in both sexes and all regions, including among men of the Northern region aged 90 years or older, differently from that observed in Table 1. The differences of percentages between the regions, which are small in the younger age groups, increase with age for both sexes (Figure 1). The greatest increases in the percentages of death due to HF among men as age increased were observed in the Southern and Northeastern regions, in this order, while for women the same was observed only in the Southern region. The lowest increases in the percentages of death due to HF among women as age increased were observed in the Southeastern and Northeastern regions.

The Southeastern region showed the lowest percentages of death due to HF among men aged 70 years and older, while, among women, that was observed earlier, from the age of 60 onwards (Figure 1).

The proportional mortality due to acute IHD, considering only the defined causes, increased with age for both sexes up to the age group of 60-69 years, in which the highest percentages were observed in all regions, and from which they began to decrease (Figure 1). Among men, the highest percentages of death due to acute IHD were observed in the Southeastern region up to the age group of 50-59 years, from which the highest percentages were observed in the Northeastern region. The Northern region had the lowest proportional mortalities due to acute IHD in both sexes up to the age group of 70-79 years, and the West-Central region showed the lowest percentages in the two oldest age groups. Among women, the Northeastern region had the highest proportional mortalities due to acute IHD in almost all age groups, except for the youngest, and the highest percentage was observed in the age group of 60-69 years.

The proportional mortality due to chronic IHD, considering only defined causes of death, increased progressively with age in the Southern and Southeastern regions among women; however, among men, it stabilized from the age group of 70-79 years onwards, with a mild decrease in the oldest age group in the Southern region (Figure 1). The Northern and Northeastern regions had the lowest proportional mortalities due to chronic IHD from the age group of 40-49 years onwards. In those two regions, the highest percentages were observed in the age group of 70-79 years in both sexes.

## Discussion

This study assessed the proportional mortalities due to HF and acute and chronic IHD as underlying causes of death in the Brazilian geoeconomic regions, stratified by sex and age. This analysis was performed for the purpose of comparison, including and excluding the occurrence of death due to IDC. It is worth noting that although proportional mortality does not represent raw mortality directly, they have an intrinsic relationship, because both share the same numerator. The difference between them lies in the denominator, which, in proportional mortality, is the total number of deaths due to any cause, considering the ill-defined deaths or not, while, in raw mortality, the denominator is the total number of individuals exposed to the risk of death. Thus, proportional mortality indicates directly the relative importance of a determined cause of death in the total set of deaths. This indicator can be preferred to that of raw mortality when the extent and quality of the death registry is more reliable than the population count. The latter, the census, is performed only once every ten years, being thus useful for only a short period of time. Regarding this study, the census was performed only in one of the years studied (2010), and, for the remaining seven years, only interpolated (2004 to 2009) and extrapolated (2011) estimates were considered. The estimation methods used by the official Brazilian Institute of Geography and Statistics (IBGE) have undergone significant changes since 2007, making the flotation of population segments, according to age and sex, important, and their use, of concerning validity.

As expected, proportional mortality due to HF^10^ increased with age increase, the highest percentages observed among women. The regional differences were more prominent in the older age groups, and even more when death certificates with IDC as underlying causes of death were excluded. In the Northern region, which had the highest percentages of IDC, considering all death certificates, the proportional mortality due to HF increased 14% and 16% among men and women, respectively, when IDC were excluded. At more advanced ages, when the percentages due to IDC were higher, the differences were more significant, reaching up to 40% more proportional mortality due to HF in the Northern region, for both sexes and over the age of 90 years.

It is worth noting that this study assessed the proportional mortality due to HF only when that was selected as the underlying cause of death, which, as already known,^10^ underestimates HF as a cause of death. Heart failure is more prevalent in the elderly, competing, in the selection of the underlying cause of death, with other diseases also common in more-advanced age groups. In addition, the rules determined for that selection discourage the coder to select HF as the underlying cause of death.^10-13^

The highest proportional mortalities due to acute IHD were observed in the age group of 60-69 years in all regions and both sexes, from which a more or less abrupt decrease was observed depending on the region analyzed. Women had lower proportional mortalities due to acute IHD as compared with men in almost all regions and age groups, regardless of the exclusion of IDC. The only exception was the age group of 40-49 years in the Northeastern region.

In addition, when analyzing proportional mortality due to acute IHD, the percentage of deaths due to IDC can conceal differences between regions, sexes or age groups. In the age group of 60-69 years, when IHD has its highest percentages, the greatest increases in proportional mortality due to IHD after excluding IDC were observed among women in the Northeastern region (22%) and men in the Northern region (18%).

The differences in mortality rates due to IHD between Brazilian regions have long been known,^14^ as has the difficulty of interpreting mortality data in regions with a high rate of deaths attributed to IDC. A recent study analyzing the trend of mortality rates due to IHD in Brazil from 2000 to 2010 has evidenced outstanding regional differences, which, according to the authors, can be explained by differences in socioeconomic conditions and health care structures.^15^

This is more easily perceived when an improvement in socioeconomic indicators precedes the reduction in mortality due to DCS, and there is a strong correlation between the progression of those indicators and the drop in mortality.^16^

One measure of the quality of information on mortality is the percentage of causes of death selected as ill defined, comprised in chapter XVIII of the CID-10.^1^ In Brazil, in 2003, the underlying cause of 13.3% of the deaths was encoded as ill-defined, the highest percentages found in the Northern and Northeastern regions.^17^ The percentage found in this study was lower, with IDC selected as the underlying cause of death in 8.3% of all deaths. This seems to indicate an improvement in the quality of information of death certificates, because of the reduction in the percentage of deaths due to IDC. In fact, this study shows that, in Brazil, the proportional mortality due to IDC decreased progressively from 12.3%, in 2004, to 6.7%, in 2011. 

It is worth noting that a recent study assessing the behavior over time of mortality due to IDC in Brazil and its regions, from 1996 to 2011, has reported a drop in the raw and standardized mortality rates due to IDC in all regions; in the Northern and Northeastern regions, which have the highest rates, that drop has been steeper since 2004, which can represent greater care with the accuracy of death registries. Once again, this study confirms that, from 2004 to 2011, proportional mortalities due to IDC decreased from 20.8% to 11.0% in the Northern region, and from 23.7% to 7.7% in the Northeastern region. The other regions have also shown a decline, although less prominent, because the initial rates were lower.

In a publication discussing the reality of HF in Latin America, the authors have concluded that the region is under the awful paradox of HF risk factors and HF epidemiology of developed countries, with an added high prevalence of systemic arterial hypertension, Chagas' disease and rheumatic fever, together with a lower total expenditure on health per capita.^18^

Differences between the regions regarding proportional mortality due to chronic IHD showed no important change, mainly because it was lower as compared with those due to the other causes studied, HF and acute IHD.

The proportional mortality due to IDC increased with age in all regions. The Northern and Northeastern regions maintained the leading position, reaching elevated values at the age group of 90 years or older, corresponding to one of every three death certificates in men, and one of every four death certificates in women. This jeopardizes the analysis of mortality in those regions, especially in the elderly population (Tables 1 and 2). Even excluding deaths due to IDC, such as in the proportional mortalities shown in Figure 1, one cannot state that the proportional mortality due to HF and IHD remains constant among the IDC in all age groups and for both sexes. It seems reasonable to suppose that, among the oldest individuals, the proportion of deaths that could have been attributed to HF or chronic IHD, for example those classified as ill-defined due to lack of proper information, would be higher than that among younger individuals. If this hypothesis is confirmed, proportional mortalities due to HF, for example, should be higher than those shown in Figure 1, especially among the oldest individuals, and even more prominent in those regions with the highest percentage of death due to IDC.

Data on mortality lose quality in the presence of a considerable proportion of IDC of death. Although the percentage of mortality due to IDC in Brazil decreased in past years, indicating an improvement in the quality of statistics on mortality,^8^ figures as those observed in the Northern and Northeastern regions are still concerning, and can be related to access to medical care and its quality, in addition to the proper completion of death certificates.

One limitation of this study derives from choosing the death certificate as the subject of study, because it depends on the quality of the registration and accuracy of the diagnoses made by physicians. Conversely, that is the most comprehensive source of information on deaths, because the death certificate is legally required to provide a proper destination of the deceased. Another study limitation was the use of the underlying cause of death to assess mortality, which, under certain circumstances, such as HF, can underestimate its relative importance when multiple causes, which contemplate all the causes mentioned on the death certificate, are not considered. Thus, the simultaneous presence of acute IHD and HF in the same death certificate can lead to the selection of IHD as the underlying cause of death.^19^ Therefore, the contribution of the causes assessed in this study, HF and IHD, will have to be analyzed, considering the existence of multiple causes de death.

## Conclusion

For now, considering the underlying causes of death, proportional mortality due to HF increases as age does, and, in the elderly, the highest percentages are observed among women. Proportional mortality due to acute IHD is usually higher among men, and, for both sexes, the age group of 60-69 years shows the highest values. Despite regional differences in proportional mortality due to acute IHD, it predominated among men in the age group of 40-49 years in the Southeastern region and in older age groups in the Northeastern region. Among women, proportional mortality due to acute IHD predominated from the fifth decade of life onwards in the Northeastern region.
